# AFM-Based Method for Measurement of Normal and Osteoarthritic Human Articular Cartilage Surface Roughness

**DOI:** 10.3390/ma13102302

**Published:** 2020-05-16

**Authors:** Mikhail Ihnatouski, Jolanta Pauk, Dmitrij Karev, Boris Karev

**Affiliations:** 1 Scientific and Research Department, Yanka Kupala State University of Grodno, Grodno, Ozheshko str., 22, 230023 Grodno, Belarus; mii_by@mail.ru; 2 Biomedical Engineering Institute, Bialystok University of Technology, Wiejska 45A, 15-351 Bialystok, Poland; 3 Department of Traumatology, Orthopedics and Field Surgery, Grodno State Medical University, Gorkogo str. 80, 230009 Grodno, Belarus; dmitriy.karev@gmail.com; 4 Department of Orthopedic and Traumatology, Grodno City Emergency Hospital, Sovietskih Pogranichnikov str., 115, 230027 Grodno, Belarus; bkarev@gmail.com

**Keywords:** atomic force microscopy, hyaline cartilage, osteoarthrosis, surface roughness

## Abstract

In osteoarthrosis, pathological features of articular cartilage are associated with degeneration and nanomechanical changes. The aim of this paper is to show that indentation-atomic force microscopy can monitor wear-related biomechanical changes in the hip joint of patients with osteoarthritis. Fifty patients (N = 50), aged 40 to 65, were included in the study. The mechanical properties and the submicron surface morphology of hyaline cartilage were investigated using atomic force microscopy. Measurements of the roughness parameters of cartilage surfaces were performed, including the arithmetic average of absolute values (*Ra*), the maximum peak height (*Rp*), and the mean spacing between local peaks (*S*). The arithmetic mean of the absolute values of the height of healthy cartilage was 86 nm, while wear began at *Ra* = 73 nm. The maximum changes of values of the roughness parameters differed from the healthy ones by 71%, 80%, and 51% for *Ra*, *Rp*, and *S*, respectively. Young’s modulus for healthy cartilage surfaces ranged from 1.7 to 0.5 MPa. For the three stages of cartilage wear, Young’s modulus increased, and then it approached the maximum value and decreased. AFM seems to be a powerful tool for surface analysis of biological samples as it enables indentation measurements in addition to imaging.

## 1. Introduction

Articular cartilage is the connective tissue of diarthrodial joints and plays specific biomechanical functions, i.e., lubrication, load-bearing, and energy dissipation [1,2]. Healthy articular cartilage consists of a small number of cells (chondrocytes), water (within a range from 66% to 80%), solid components (20%–34%) including 5%–6% of hydroxyapatite, type-II collagen (48%–62%), and proteoglycan (22%–38%) [3,4,5,6]. Pathological features of articular cartilage are associated with its degeneration, surface morphology, and biomechanical changes in its nanoscale structure, and may result in osteoarthritis. Moreover, increased synthesis of collagen, non-collagen proteins, proteoglycans, hyaluronate, and desoxyribonucleic acid can be detected in its deeper layers [7]. Osteoarthrosis (OA) is a substantial public health problem. The prevalence of OA increases with age; the highest incidence is observed among women over 45 years old [8]. According to the general classification of the American Society of Rheumatology, osteoarthritis ranks 4th among all the rheumatic diseases, affecting 80% of the European population aged 75 years and older [9]; among adults 60 years of age or older, over 10% of men and 13% of women in the United States suffer from OA [10]. The aetiology of osteoarthrosis is unclear; however, the risk factors include age, gender and hormones, genetic factors, race/ethnicity, congenital/development conditions, diet, obesity, injuries, occupation, physical activity, alignment, mechanical factors, and laxity [11,12,13,14]. The disease is characterized by an imbalance between anabolic and catabolic processes in joint tissues, loss of articular cartilage, and synovial inflammation [11,12]. At the initial stage, it may remain chronic for several years, without any symptoms. During its development, OA may affect all joints, muscles, as well as all articulating tissues and menisci in the knee [8] with symptoms such as pain or stiffness in the joint, followed by deformation and reduced mobility. 

The biomechanical properties of cartilage are crucial for the functionality of the hip joint. The surface topographies of the hip joint have been studied using the most common imaging modalities: radiography [15,16], atomic force microscopy [17,18,19,20], computed tomography (CT) [21], ultrasound [22], MRI and nuclear medicine [23], as well as others [24]. Radiography has been considered as the standard technique in defining rheumatic disorders; it is useful in detecting structural bone abnormalities in late-stage OA. However, it cannot detect changes in features of the disease around the joint [16,25]. Therefore, magnetic resonance imaging (MRI) is a more suitable tool as it shows pathological features related to the severity of OA and allows 3D visualization of articular cartilage [26]. In addition, computed tomography (CR) shows the affected soft tissue; however, compared to MRI, the assessment of soft tissue structures is limited. Although CT arthrography is invasive, it can be used in the evaluation of focal cartilage defects [25]. Another technique is the ultrasound (US), which enables detection of changes in cartilage and other soft tissues of the joint in the early and late stages of osteoarthritis. The imaging technique is commonly regarded as one of the most useful ones due to its low cost and repeatability [27,28]. Understanding the structural and mechanical characteristics of healthy and osteoarthritic cartilage requires the use of technologies at a micron scale. Additionally, it has been proved that morphological and biomechanical changes that occur at the onset of OA should be visualized at the nanometer scale [29]. 

The last several years have seen the development of nanostructural imaging modalities. These include atomic force microscopy (AFM), scanning electron microscopy (SEM), confocal microscopy (LSCM), and laser scanning; they are used to examine cartilage surfaces in three dimensions at micron or submicron resolutions [30,31,32,33,34,35,36,37]. In [1,38,39,40,41], the authors used AFM to study bovine, porcine, and ovine cartilage surfaces. For the analysis, the arithmetic average of absolute values (*Ra*) and the maximum peak height (*Rp*) were used. These parameters play a crucial role in the study of pathological features at the nanometer scale and may signal biological activity resulting from OA. Additionally, animal cartilage can be used as a model system for human cartilage due to the fact that animal tissue enables a suitable assessment of the biomechanical properties of the hip. The main problem, however, is the difference in shape, size, biochemical content, and matrix architecture [37], compared to human cartilage. These particular biomechanical properties of human cartilage are crucial in understanding the progression of OA. Human cells are usually obtained post-mortem during an autopsy; it is challenging to obtain samples from healthy living cells, so their biomechanical features are not well recognized. Hence, the aim of this paper is twofold. First, quantitative techniques for the study of both healthy and osteoarthritic living cells of articular cartilage at the micron scale are developed. Then, AFM microscopy is shown to be able to monitor wear by OA-related biomechanical changes in the hip joint in patients at the different stages of osteoarthritis.

## 2. Methods

Experiments on human subjects were performed in accordance with internationally accredited guidelines, and had been approved by the Ministry of Health of the Republic of Belarus (No. 3, 13/01/2017). The experiments were carried out in accordance with the approved guidelines. Informed written consent to perform this experiment was obtained from all patients. 

### 2.1. Samples of Human Articular Cartilage

The hospitalized patients from Grodno City Emergency Hospital were male and female in equal numbers (N = 50), aged 40 to 65 years. They had undergone surgery for total endoprosthesis of the hip in connection with the diagnosis of osteoarthritis, or fracture of the neck or head of the femur. Over a period of two years (2017–2018), 500 surgical procedures were performed. An exclusion criterion in the patient selection was the diagnosis of rheumatoid arthritis or the presence of joint and bone tumors. Human articular cartilage was harvested from the femoral heads by cutting specimens off the underlying bone with a sharp razor blade, yielding ∼5 mm × 5 mm pieces, ∼5 mm thick. The cartilage was covered with a fluid interfering with the mechanical properties due to surface tension. At the same time, the cartilage was saturated with water. The drying process changes the properties of cartilage, so the level of drying was determined experimentally in order to obtain the necessary accuracy of measurement. All the tests were performed at a room temperature of 20 ± 0.5 °C and a humidity of 56 ± 1.0%. The process of specimen dehydration is described by (1):

(1) $$m=m_{0}{\left(1+t\right)}^{b}$$

where: *m* is the mass (current value of mass) of a specimen, *m*_0_ is the initial mass, coefficient, *b* = $-0.\bar{1}\bar{4}$, and *t* is time [hours].

### 2.2. Analysis of Submicron Surface Morphology in Humans 

**Figure 1:**
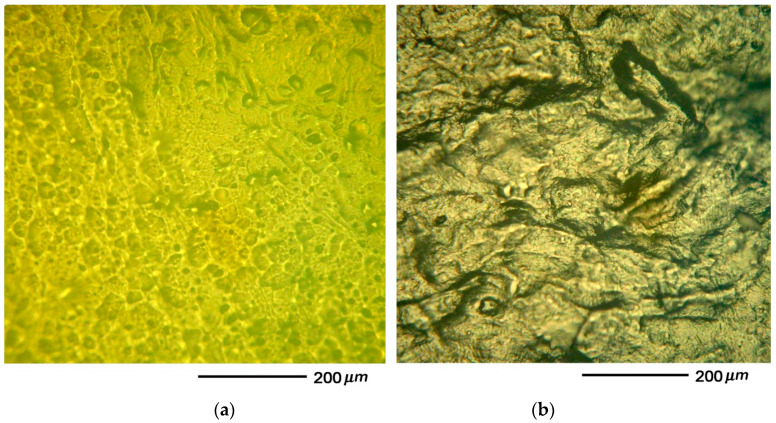
Optical images 200× of human cartilage surfaces: (**a**) healthy and (**b**) affected by osteoarthritis.

The human specimens were fixed on a rigid substrate and stuck to microscope slides. Human cartilage specimens (N = 500) were selected based on initial visual assessment of surface degradation at optical magnifications of 100×, 200×, and 500×. The images were obtained in reflected light using a ©Micro 200T-01 optical microscope (Planar JSC, Minsk, Belarus). Undamaged (healthy) human cartilage specimens and those affected by *osteoarthritis* are presented in Figure 1a,b, respectively. Tests of submicron surface morphology of human specimens were performed using AFM NT-206 (©MicroTestMachines, Gomel, Belarus) in the static scanning mode. A CSC38 MikroMasch^®^ silicon probe (MikroMasch, Watsonville, CA, USA) was used: the resulting tip radius was less than 35 nm, the full tip cone angle was 40°, the total tip height was 12 to 18 µm, the probe material was n-type silicon, and a type A cantilever was used. The resonance frequency of the cantilever ranged from 8 to 32 kHz, the force constant was 0.01 to 0.36 N/m, length: 250 ± 5 µm, width: 32.5 ± 3 µm, and thickness: 1.0 ± 0.5 µm. The areas of specimens without surface waviness or high strain amplitudes were found prior to scanning or indenting by means of an optical microscope built into the AFM. The results of scanning were classified at three scales: at low magnification with a scan area of *Ar* = 18 × 18 µm^2^, at medium magnification with a scan area of *Ar* = 9 × 9 µm^2^, and at large magnification with a scan area of *Ar* = 3 × 3 µm^2^. The degree of surface wear was determined by measuring the roughness parameters. The SurfaceExplorer (©MicroTestMachines, ver 1.1.5, MicroTestMachines, Gomel, Belarus) software and nano images (ver 6.128.15, Mikhail Ihnatouski, Grodno, Belarus) were used to visualize the experimental data and to measure the roughness parameters.

Specimens obtained after division were used for further studies of submicron surface morphology and mechanical properties. Measurements of the roughness parameters of cartilage surfaces were performed, including the arithmetic average of absolute values (*Ra*), the maximum peak height (*Rp*), and the mean spacing between local peaks (*S*). The roughness parameters were measured at 5 points of each of the 25 specimens for three different values of *Ar*.

### 2.3. Analysis of the Mechanical Properties of Human Cartilage

**Figure 2:**
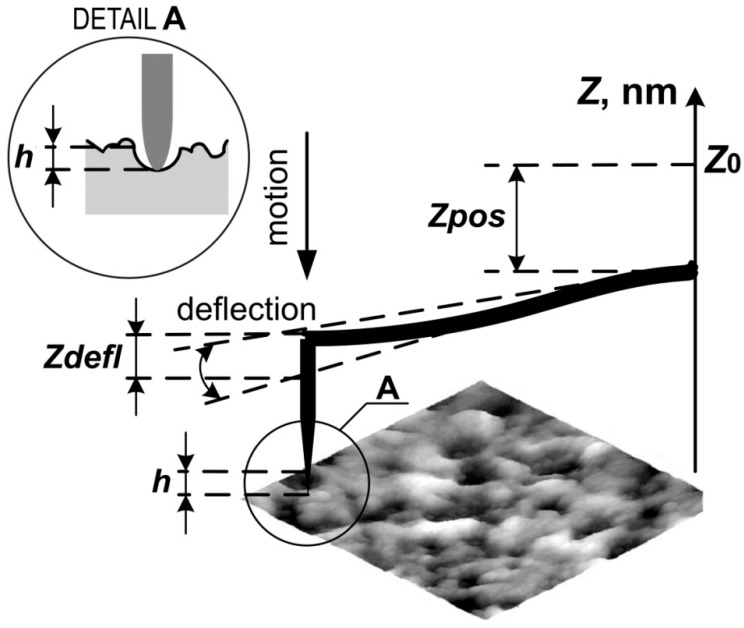
Scheme of AFM indentation: $Z_{0}-\mathrm{the}\;\mathrm{starting}\;\mathrm{point}\;\mathrm{of}\;\mathrm{the}\;\mathrm{console};\;\;$ $Z_{defl}-$ the bend of the console$;\;Z_{pos}-$ the displacement of the console along the vertical axis; *h* - the penetration of the probe into cartilage.

The radius of the AFM tip and the stiffness of cantilevers were calibrated. AFM was used to measure the mechanical properties of human cartilage specimens. The measured quantities were the bend of the console $\left(Z_{defl}\right)\;$and the displacement of the console along the vertical axis $\left(Z_{pos}\right)$. The penetration of the probe into cartilage (Figure 2) is presented as (2) [42]:

(2) $$h=Z_{pos}-Z_{defl}$$

Young’s modulus (*E*) at a point on the surface can be calculated as follows:

(3) $$E=\frac{3}{4}\left(1-V^{2}\right)\frac{k}{R^{1/2}}\frac{Z_{defl}}{{\left(Z_{pos}-Z_{defl}\right)}^{2/3}}$$

where: *V* = 0.5 is the Poisson’s ratio of the cartilage, *k* = 0.08 N/m is the stiffness of the cantilever of CSC 38, and *R* = 30 nm is the radius of the needle-point of the tip of CSC 38. Thus, the results of the measurements were the relationship between Young’s modulus and the depth of penetration into the surface (E(h)).

The surface of each sample was indented using AFM in the course of three measurements through the introduction of an indenter at a selected point on the surface. The obtained values of Young’s modulus were averaged. Cross-sections of human cartilage specimens were sequenced twice with a 100 µm stem parallel to the initial surface. The surfaces of the cross-sections were indented sequentially using AFM.

### 2.4. Statistical Analysis

Statistical analyses were performed using the Statistica software (StatSoft 13.1, Cracow, Poland). One-way analysis of variance was used to identify the degree of dehydration of specimens. A *p*-value < 0.05 was considered as statistically significant. The data were presented as the mean ± standard deviation or min ÷ max.

## 3. Results 

### 3.1. Analysis of Submicron Surface Morphology of Human Cartilage

**Figure 3:**
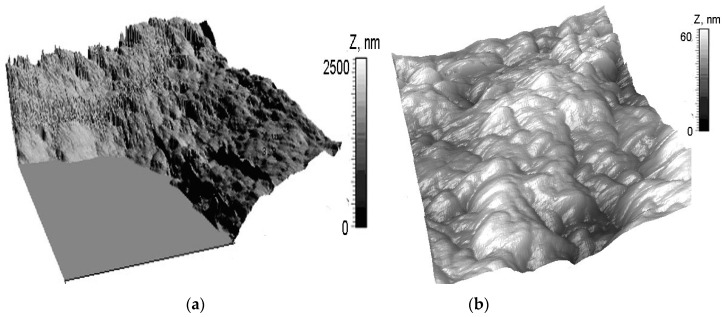
AFM images of a healthy human surface: (**a**) 9 × 9 μm^2^, *Z*max = 2500 nm, and (**b**) 3 × 3 μm^2^, Zmax = 60 nm.

Preliminary series of measurements were carried out to identify the degree of dehydration of specimens, ensuring stable repeatability of measurement results. One hundred and forty specimens (N = 140) were taken without prior control of the degree of wear. It was found that measurement results became statistically reliable (*p* < 0.05) after 12 hours, when the specimens had lost 30% of the liquid. Morphology of the areas of human cartilage not affected by osteoarthritis were investigated. Non-homogeneous morphological structures, including submicronic protrusions and depressions (lacunae), were found on the cartilage surface, without macroscopic destruction. Many of the lacunae were over 2 µm deep. Forty-eight elliptical lacunae with similar depths were found on the surfaces of ten specimens. The complex structure of near-surface collagen fibers was clearly visible in AFM images. The diameters of collagen fibers ranged from 50 to 350 nm (Figure 3).

**Figure 4:**
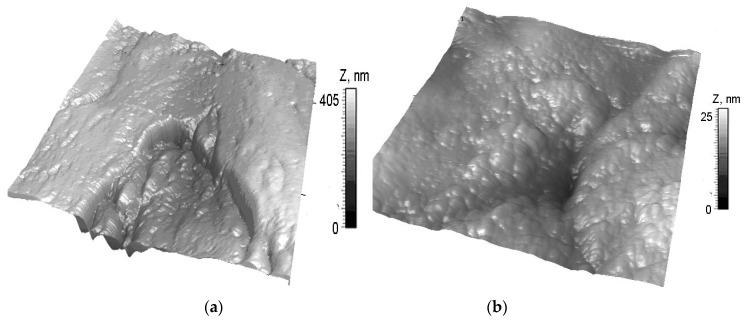
AFM images of a worn human surface: (**a**) 9 × 9 μm^2^, Zmax = 405 nm, and (**b**) 3 × 3 μm^2^, Zmax = 25 nm.

Those human cartilage surfaces that were affected by osteoarthritis to a larger degree had a weaker set of morphological structures visible in AFM images (Figure 4). The surfaces of the specimens had irregularities that were less noticeable compared to surfaces of healthy cartilage (Figure 3). The lacunae found on the surfaces of cartilage affected by osteoarthritis were less than 2 µm deep, which enabled measurements with the use of AFM (Figure 4a,b). Twenty-three lacunae with a thickness of approx. 500 nm were found on the surfaces of ten specimens.

The roughness parameters were measured at 5 points of each of the specimens for three different values of *Ar*. Three hundred and seventy-five measurements (*N* = 375) of the roughness parameters of twenty-five healthy human cartilage specimens, and one thousand one hundred and twenty-five (*N* = 1125) measurements of seventy-five specimens affected by osteoarthritis were performed. In Table 1, the roughness parameters of healthy cartilage are presented as the arithmetic mean and the standard deviation (std). The measurement results constitute the starting points on the wear scale.

The size of change (min ÷ max) of the roughness parameters of cartilage affected by osteoarthritis is presented in Table 2. For the purpose of further analysis, the values measured at *Ar* = 9 × 9 were used. 

Due to the fact that human cartilage samples were divided into two groups with the use of optical microscopy, i.e., healthy ones and those affected by osteoarthritis, a significant gap on the wear scale was obtained. As expected, cartilage wear resulting from an illness is characterized by a continuous consistency, ranging from larger to smaller values of the arithmetic mean of absolute height values (*Ra*). The continuity and monotony of the changes in the maximum peak height (*Rp*) and the mean spacing between local peaks (*S*) are not reflections of the same trivial fact. The arithmetic mean of the absolute values of the height of healthy cartilage was 86 nm, while wear began at *Ra* = 73 nm. The values of the maximum changes of the roughness parameters differed from the healthy ones by 71%, 80%, and 51% (*p* < 0.05) for *Ra*, *Rp,* and *S*, respectively. The obtained data concerning the wear parameters of cartilage surfaces make it possible to determine the impact of the mechanical properties of surface on their wear. The seventy-five specimens of surface affected by osteoarthritis were divided into three groups: small, medium, and heavily affected by osteoarthritis, using the method of partitioning *Ra* = 25.0 ÷ 61.0 nm into three subranges.

### 3.2. Analysis of the Mechanical Properties of Human Cartilage

**Figure 5:**
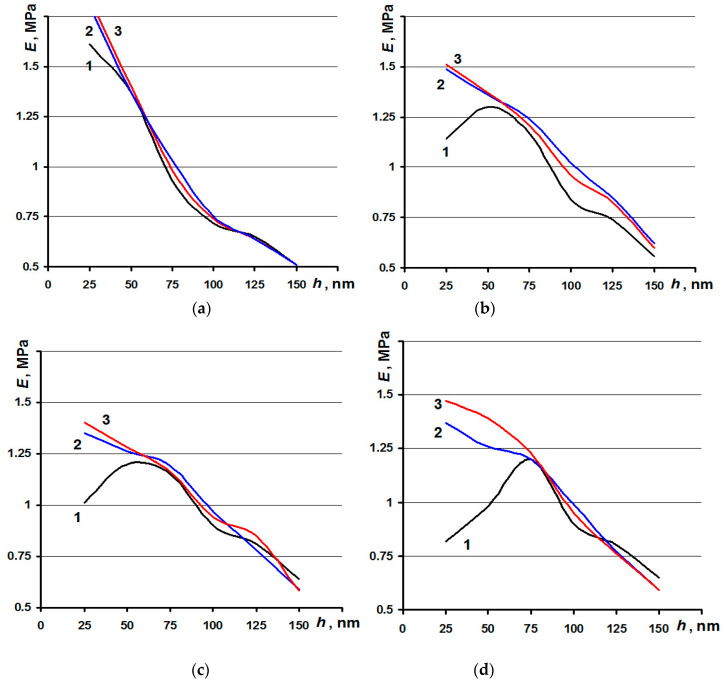
Relationships between Young’s modulus and the indentation depth: (**a**) healthy, (**b**–**d**) three stages of wear by osteoarthrosis (OA): (**b**) 49.0 nm < *Ra* < 61.2 nm; **c**) 36.8 nm < *Ra* < 49.0 nm; and (**d**) 24.6 nm < *Ra* < 36.8 nm): (1) cartilage surface layer; (2 and 3) subsurface layers after the first and second cross-sections.

The relationship between the mechanical properties of human cartilage and the depth of penetration (E(h)) into cartilage (*Ra* ≈ 85.8 nm) at different stages of wear (49.0 nm < *Ra* < 61.2 nm, 36.8 nm < *Ra* < 49.0 nm, and 24.6 nm < *Ra* < 36.8 nm, measured at *Ar* = 9 × 9 µm^2^) is presented in Figure 5a–d. Lines marked 1 concern the surface layer of cartilage. Lines marked 2 and 3 concern the cross-sections in the subsurface layer (cartilage tissue). Young’s modulus of healthy cartilage surfaces ranges from 1.7 to 0.5 MPa (Figure 5a, line 1). For the three stages of wear, Young’s modulus first increases for all *Ra* ranges (from 1.14 to 1.3 MPa, from 1.02 to 1.2 MPa, and from 0.82 to 1.2 MPa), and then it approaches the maximum before decreasing (Figure 5b–d, line 1). The relationships between Young’s modulus and the depth of indentation into the cross-sections of both healthy cartilage and that affected by osteoarthritis are similar. Young’s modulus decreases monotonically with increasing intensity at all three stages of wear (from 1.7 to 0.5 MPa, from 1.2 to 0.65 MPa, from 1.0 to 0.6 MPa, and from 0.8 to 0.6 MPa) (Figure 5a–d, line 2,3).

**Figure 6:**
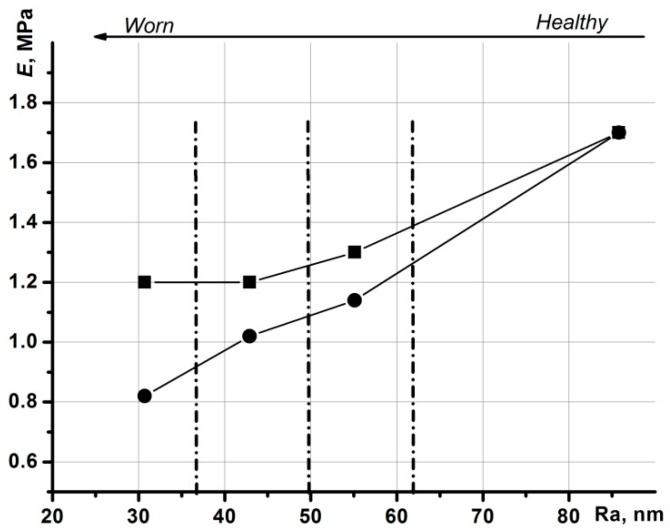
Relationships concerning Young’s modulus of cartilage wear: round dots represent the values of Young’s modulus on the surface; square dots represent the maximum values of Young’s modulus.

Figure 6 shows the impact of the mechanical properties on cartilage wear. The dash-dotted lines divide the axis of wear into three subranges. 

The points in Figure 6 were obtained from lines marked as 1 in Figure 5a–d. The round dots in Figure 6 are Young’s modulus on the surface, while the square dots are the maximum values of Young’s modulus. The maximum value of Young’s modulus and its value on the surface coincided with healthy cartilage.

## 4. Discussion

The current trend in science is to develop methods of measurement of surface roughness of human articular cartilage in various joint diseases, osteoarthritis being the most common rheumatic disease and cause of disability. The authors investigated changes in the mechanical properties and the surface roughness of articular cartilage in patients with osteoarthrosis of the hip joint and compared the results to living cells of healthy cartilage at the μm scale, which is comparable to the sizes of matrix molecules and cells [43]. Specimens of healthy cartilage surfaces are usually taken post-mortem during an autopsy. However, they may display symptoms of osteoarthritis. Moreover, fatal diseases may also be a cause of hidden cartilage pathologies. A challenge is the assessment of both healthy and osteoarthritic living cells of articular cartilage at a micron scale. Some studies of pathological changes of degenerated cartilage have been carried out, but mostly on animals, e.g., mice, pigs, or sheep [1,17,44,45,46]. OA progression in animals was similar to the biomechanical properties of the human hip, based on the histological analysis presented in [47]. In [48], the authors compared the material properties of healthy human hip cartilage to the acetabulum of baboons, dogs, and bovines. The results showed significant topographical variations of articular cartilage as well as of the mechanical properties of hip cartilage among the four species. Human hip cartilage was the stiffest in all the test sites, whereas bovine tissue was the softest. The authors stated that human tissue had the smallest Poisson’s ratio and permeability. From the anatomical point of view, canine and baboon hips had similar characteristics to the human hip joint. However, the baboon represented the most appropriate animal model of healthy human hip articular cartilage [48].

In this study, cartilage samples were received during endoprosthesis removal in an equal number of women and men aged 40 to 65 years (median: 58 years). With the aim of finding areas affected by osteoarthritis, it was assumed that the main symptom of osteoarthritis was a degenerative-dystrophic change in the surface. The orientation of collagen fibers is determined by the direction of the force lines that arise during cartilage deformation; a loss of matrix glycosaminoglycans, mainly in the surface and the intermediate zones, is a characteristic sign of cartilage destruction [42]. The AFM contact method was used to expose the internal sections of the tissue. A microspherical or a pyramidal tip is usually programmed to indent the sample tissue cells to a preset force or depth. The Hertz model was used for the evaluation of Young’s modulus of cells and tissues using the force spectroscopy mode by fitting the loading portion of each indentation force to the depth curve [20,49]. The force curve is obtained by recording the cantilever deflection as the tip is brought into contact with the surface and then retracted. The indented sample is assumed to be extremely thick in comparison with the indentation depth [50]. Although the indentation depth in the case of AFM probing of tissue falls within the range of hundreds of nanometers, which is higher than the appropriate depth for the Hertz model, it has been shown in many studies that the Hertz model describes the experimental data [42,51] sufficiently. Although the AFM method can deform and distort the cartilage tissue [17,52], several studies have shown that this method is a credible way to evaluate the mechanical properties of the internal section of cartilage tissue [53,54]. The AFM technique consisted of two main steps. First, the cartilage surface was adequately prepared. The authors proposed Equation (1) for obtaining cartilage mass loss during dehydration. Then, the duration of dehydration necessary for obtaining stable results of measurements of the mechanical properties was determined. It was found that measurement results became statistically reliable (p < 0.05) after 12 hours, when the specimens had lost 30% of liquid. All tests were performed in the desiccator at a room temperature of 20 ± 0.5 °C and a humidity of 56 ± 1.0%. The stability of the mechanical properties after dehydration was achieved when the liquid had evaporated from the surface, but the critical drying of the cartilage had not yet begun. Secondly, topographical measurement of the prepared region was performed to optimize the surface preparation parameters.

Knowledge about the nanomechanical properties of the hip joint yields information about its function. A loss of the biomechanical function may be a symptom of biochemical changes that occur during the onset and progression of OA. Mechanical properties of human cartilage were measured at *Ar* = 9 × 9, due to the fact that at lower magnifications, deeper troughs were not visible. Highly degenerated cartilage surfaces had a weaker set of morphological structures in AFM images. The results show differences in the nanomechanical properties of OA samples compared to healthy ones. It was found that specific surface features allowed us to identify the evolution of osteoarthritis at the micrometer scale; these features were the arithmetic average of absolute values (*Ra*), the maximum peak height (*Rp*), and the mean spacing between local peaks (*S*). *Ra* and *Sa* are two- and three-dimensional amplitude parameters for determining the value of roughness, respectively. Although some studies have reported that two-dimensional parameters fail to describe significant changes in the surface morphology [55,56], the results of this study show that those parameters can be applied in the assessment of the progression of OA development. The results in question demonstrated incremental heterogeneity in the nanomechanical properties in the course of OA development. This is in agreement with [13,57], where the authors stated that OA manifests as a heterogeneous disease with varying clinical features and biochemical characteristics. A significant decrease in both chondroitin sulfate and keratan sulfate was combined with dystrophic and necrotic changes in chondrocytes as well as an increase in the number of deformed lacunae. At the submicron level, the authors of this study proved that the spatial surface features differed between the three osteoarthritis grades. 

Cartilage experiences dynamic loads, which is why the mechanical properties of healthy cartilage should provide it with an ability to change its geometric shape and absorb the load. Moreover, it should quickly restore its geometric shape after unloading. In cartilage affected by osteoarthritis, Young’s modulus decreases, which means that the abilities for depreciation and restoration of the initial geometric shape deteriorate. A unique nanomechanical profile was found for both healthy and osteoarthritic human hip joint tissue. Young’s modulus of healthy cartilage surfaces ranged from 1.7 to 0.5 MPa, whereas for the three stages of wear by OA, the values were as follows: (1.14 to 1.3 MPa), (1.02 to 1.2 MPa), and (0.82 to 1.2 MPa). These data were not significantly dependent on age and gender (*p* > 0.05). In addition, our studies have shown uneven changes in Young’s modulus at depth as well as the various surface and subsurface layers of cartilage worn by osteoarthritis. Young’s modulus decreased monotonically with increasing intensity in all three stages of wear. AFM revealed a decrease in the values of Young’s modulus (up to 100 nm) of the upper layer of hyaline cartilage and an impact of its reduction on the degree of wear. However, the relationships between Young’s modulus and the depth of indentation into the cross-sections of both healthy cartilage and cartilage affected by osteoarthritis are similar. In the future, the mechanical properties of cartilage layers deeper than 100 nm (cross-sections) should be studied. This study is in agreement with [58], where atomic force microscopy (AFM) was used to determine the effective indentation modulus and to measure the surface morphology of moist cartilage surfaces. The study found that the mean values of the practical indentation modulus of worn cartilage were lower than those of healthy cartilage, used as the control sample. A medium-to-strong correlation between the useful values of the indentation modulus and the OA grades was found. The relationship between surface topography and the useful values of the indentation modulus of the cartilage surfaces with OA progression was weakly correlated [58].

In this study, the cartilage was obtained from people who had a clinically established diagnosis of osteoarthrosis. The cartilage surface areas were divided into healthy ones and those with different degrees of wear by OA. It was found that AFM for the articular cartilage was correlated to the findings of morphologic imaging in patients with clinical symptoms of osteoarthritis. The results of this study might indicate the potential of AFM to quantify those pathological changes in conditions for cartilage that alter the biomechanical properties of the hip joint. There can be numerous applications of the proposed method. First, the AFM technique makes it possible to examine donor cartilage for transplantation in many of its areas, as it requires the removal of a minimum amount of cartilage. Moreover, the presented method can be implemented to investigate the practical values of the indentation modulus of clinical osteoarthritic cartilage and to assist in the understanding and assessment of OA. Finally, the AFM methodology may be used for the study of the mechanical properties of cartilage samples extracted from the joint by laparoscopy during non-total surgical operations. What is more, prediction of the future course of osteoarthritis and the patient can be made on the basis of AFM studies.

The limitation of this study is the small scanning area (~100 µm^2^) for measuring the cartilage roughness. However, to the best of the authors’ knowledge, this is the first study assessing the mechanical properties and the submicron surface morphology of human hyaline cartilage based on samples of healthy living cells. Joint replacement is becoming a routine surgical operation. Nowadays, the development of supportive therapy allows to improve the quality of the patient’s life. In the future, the authors will analyze the impact of supportive care on postponing the necessity for a hip endoprosthesis in patients with osteoarthritis. Both AFM and confocal microscopy will be used in the new study; both methods combined will lead to exciting research results.

## 5. Conclusions

The current trend in science is to develop methods of measurement of the surface roughness of human articular cartilage in different joint diseases. AFM seems to constitute a powerful tool for the surface analysis of biological samples based on indentation measurements and imaging. The quantitative characterization of structural changes in the cartilage surface could be used for understanding the disease progression and for developing objective OA assessment criteria. Such a method could be used as a convenient screening tool complementary to the conventional histological analysis.

## Figures and Tables

**Table 1:** Mean (SD) roughness parameters of healthy human surfaces (N = 357).

*Ar* [µm^2^]	*Ra* [nm]	*Rp* [nm]	*S* [nm]
18 × 18	96 (6)	526 (18)	953 (13)
9 × 9	86 (5)	436 (13)	816 (11)
3 × 3	28 (4)	147 (11)	239 (12)

**Table 2:** The size of change (min ÷ max) of the roughness parameters affected by osteoarthritis (N = 1125).

*Ar* [µm^2^]	*Ra* [nm]	*Rp* [nm]	*S* [nm]
18 × 18	58 ÷ 73	177 ÷ 353	567 ÷ 661
9 × 9	25 ÷ 61	86 ÷ 145	403 ÷ 551
3 × 3	9 ÷ 12	38 ÷ 64	235 ÷ 298
